# Efficacy and feasibility of catheter ablation in elderly patients with atrial fibrillation

**DOI:** 10.1002/clc.24195

**Published:** 2023-11-16

**Authors:** Naoya Kataoka, Teruhiko Imamura

**Affiliations:** ^1^ Second Department of Internal Medicine University of Toyama Toyama Japan

To the Editor

The number of elderly patients with multiple comorbidities, including atrial fibrillation, is increasing so far. The impact of catheter ablation for atrial fibrillation on elderly patients with atrial fibrillation remains uncertain. Aldaas and colleagues demonstrated that catheter ablation for atrial fibrillation was similarly effective and feasible in patients aged 80 years or older when compared with a younger cohort.^1^ Several concerns have been raised.

Elderly patients had a significantly higher cumulative incidence of all‐cause death/hospitalization after catheter ablation in their unadjusted comparative analyses.^1^ This is not surprising as elderly patients have multiple comorbidities and more advanced sarcopenia than the younger cohort. These worse baseline characteristics may have a negative prognostic impact, regardless of the correction of atrial fibrillation. The authors also adjusted for potential confounders, and the clinical outcomes of older patients were as good as those of younger patients.^1^ However, the unadjusted time‐to‐event analyses may be more representative of real‐world clinical practice.

One of the procedural parameters missing from their study is the energy source.^1^ How many patients underwent radiofrequency ablation and cryoballoon ablation, respectively? Efficacy and feasibility seem to be comparable between the two energy sources.^2^ However, the applicability of such a general consensus to the elderly cohort remains unknown.

Many elderly patients have frailty, including not only physical but also mental ones. Patients with arrhythmias have more advanced depression than others. Aggressive catheter ablation may improve mental frailty in elderly patients with atrial fibrillation.^3^ Did the authors assess patients' quality of life and mental status before and after catheter ablation?

## CONFLICT OF INTEREST STATEMENT

The authors declare no conflict of interest.

## Data Availability

NA.
